# The Role of Myeloid Populations during Perinatal Liver Injury and Repair

**DOI:** 10.33696/immunology.3.076

**Published:** 2021

**Authors:** Anas Alkhani, Sarah Mohamedaly, Amar Nijagal

**Affiliations:** 1 Department of Surgery, University of California, San Francisco, CA, USA; 2 Liver Center, University of California, San Francisco, CA, USA

**Keywords:** Perinatal liver inflammation, Extramedullary hematopoiesis, Biliary atresia

## Abstract

Perinatal liver inflammation can have life-threatening consequences, particularly in infants and young children. An example of a hepatic inflammatory disease during infancy is biliary atresia (BA), an obliterative cholangiopathy that rapidly progresses to hepatic fibrosis and liver failure. The aggressive nature of BA in neonates compared to the pathogenesis of inflammatory liver diseases in adults, suggests that the mechanisms responsible for restoring tissue homeostasis following inflammation are impaired in affected infants. This article reviews our recent findings demonstrating that the relative abundance of Ly6c^Lo^ non-classical monocytes promotes resolution of perinatal liver injury in a murine model of perinatal hepatic inflammation. Our research also identifies a potential co-regulatory role between neutrophils and non-classical monocytes. Further work is needed to understand how neutrophils regulate other myeloid populations during perinatal liver inflammation. Elucidating the mechanisms that govern perinatal liver injury and repair may lead to the development of immune-directed therapies that can be used to mitigate the devastating effects of diseases like BA.

## Introduction

The maturation of the immune system is a complex process that undergoes major transitions during fetal and neonatal development [1]. Throughout this developmental window, the response to liver injury is dependent on the nature and timing of the insult [2]. For example, fetal damage following maternal infection can lead to the development of innate immune memory in the fetus, a process that may mitigate the impact of future insults [3]. It remains unknown how immune responses to injury are orchestrated and sustained during this period of hematopoietic development and immune system maturation.

In our recent study, “Ly6c^Lo^ non-classical monocytes promote resolution of rhesus rotavirus-mediated perinatal hepatic inflammation,” we demonstrate the importance of monocyte subsets in mitigating inflammatory insults to the perinatal liver. Using a rhesus rotavirus (RRV) model of perinatal liver injury, we investigated changes in composition of the hepatic myeloid immune compartment in late-gestation fetuses and in neonatal pups [4]. Our initial experiments demonstrate that the late-gestation fetus is resistant to liver inflammation, while neonatal pups develop severe liver disease. We attributed the resistance to inflammation in the late-gestation fetus to the physiological abundance of Ly6c^Lo^ non-classical monocytes observed at this time. Since Ly6c^Lo^ non-classical monocytes exert anti-inflammatory and pro-reparative functions [5–7], we hypothesized that the abundance of these cells plays a role in rendering the fetus resistant to RRV-mediated liver injury.

To test this hypothesis, we manipulated the myeloid compartment within the neonatal liver to resemble the fetal environment, thereby establishing an abundance of Ly6c^Lo^ non-classical monocytes. Using anti-Ly6g targeted neutrophil depletion [8], and anti-CCR2 targeted Ly6c^Hi^ classical monocyte depletion [9], we expanded the number of Ly6c^Lo^ non-classical monocytes within RRV-injected neonatal livers. Importantly, antibody-mediated depletion of neutrophils and Ly6c^Hi^ classical monocytes increased both the percentage and the absolute number of Ly6c^Lo^ non-classical monocytes, indicating both a proportional and numerical expansion of these cells. Furthermore, the rise in Ly6c^Lo^ monocytes was associated with disease resolution in the neonate, supporting the idea that Ly6c^Lo^ non-classical monocytes play a role in resistance to perinatal liver injury and resolution of disease. To confirm Ly6c^Lo^ non-classical monocytes are responsible for disease resolution and resistance [4], we used a Cx3cr1 small molecule inhibitor (AZD8797) to selectively block Ly6c^Lo^ non-classical monocyte function in the setting of neutrophil and classical monocyte depletion [10–12]. Blockade using this small moleculae inhibitor restored susceptibility to RRV-mediated injury, supporting the idea that Ly6c^Lo^ non-classical monocytes mitigate perinatal liver inflammation [4].

## Neutrophil-mediated Regulation of Inflammation and Tissue Repair

Recent evidence suggests that neutrophils play a vital role in regulating and orchestrating inflammation and tissue repair through their interaction with the innate and adaptive immune systems [13–16]. Several studies have suggested that neutrophil absence may result in chronic injury and sequelae by altering the homeostatic response and the recruitment of monocytes in the setting of underlying tissue injury [13,16–23]. A study published by Horckmans et al. indicates that neutrophils play an essential role in regulating outcomes after cardiac tissue injury by altering the polarization state of macrophages [16]. The authors also demonstrate that the release of neutrophil gelatinase-associated lipocalin results in enhanced macrophage pro-reparative function at the injury site following myocardial infarction. Neutrophils were also found to promote tissue repair and regeneration via secretion of oncostatin M, which regulates macrophage accumulation at the site of cardiac tissue injury [24]. In the liver, neutrophils also induce cellular and necrotic debris clearance and promote vascular repair and growth after inflammation [25–28]. Furthermore, their absence in the setting of liver inflammation and cholestatic injury was found to exacerbate the liver injury and fibrosis, which in turn suggests a vital role for neutrophils in modulating the reparative mechanism within the liver after injury [29–31]. In RRV-injected and neutrophil-depleted pups, we observed an expansion of Ly6c^Lo^ non-classical monocytes [4], suggesting that the inverse correlation between neutrophils and Ly6c^Lo^ non-classical monocytes reveals a co-regulatory relationship between these two cell populations that are important for tissue repair and healing [14].

## The Liver and Spleen Actively Participate in the Kinetics of Hematopoiesis within the Neonate

An intriguing mechanism by which neutrophils may influence other myeloid populations during inflammation may involve the regulation of myeloid precursors and hematopoietic progenitors [15]. We have observed changes to myeloid precursor populations after depletion of neutrophils during RRV-mediated inflammation (unpublished observations), prompting us to examine the role of emergency myelopoiesis role during perinatal liver injury.

The liver is known to serve as the primary hematopoietic organ throughout fetal life as it harbors the hematopoietic stem cell (HSC) niche [32]. Soon after birth in the mouse, the liver no longer serves as the primary site of hematopoiesis and its hematopoietic function is replaced by the BM, the latter establishing its permanent niche through adulthood [32–34]. However, the liver maintains relatively low levels of erythropoiesis and myelopoiesis, and retains a small population of HSCs and immune progenitors throughout adulthood [34,35]. Although the liver’s contribution to extramedullary hematopoiesis (EMH) in the setting of perinatal liver injury is not known, the liver can re-emerge as a site of EMH during immune and inflammatory damage and by doing so, contribute to the overall innate immune response [35,36].

In addition to the liver, the spleen can also contribute to the overall hematopoietic activity during neonatal development and inflammation via EMH [35,36]. However, the spleen’s preferential lineage outcome and the extent of its involvement in emergency hematopoiesis has not been determined. Splenic EMH differs within the first two weeks of life when compared to adulthood [36]. Wolber et al. have found that colonies of erythro-myeloid lineages, including myeloid forming colonies, erythroid forming colonies, and mixed erythroid/myeloid forming colonies, were detectable at large numbers in the spleen in the first two weeks of neonatal life. By adulthood, the spleen becomes more restricted to an erythroid lineage, although it still maintains minimal myeloid activity [36]. The spleen was also found to harbor myeloid progenitors that serve as precursors to Cd11b^Hi^ myeloid cells, and Ly6c^Lo^ and Ly6c^Hi^ monocytes in the setting of tissue injury and systemic disease [37,38]. These data support the idea that, in addition to the liver, the spleen may exhibit age-dependent hematopoietic activity that may govern immune responses during perinatal liver inflammation.

## Conclusion

Understanding the immune response to liver injury during perinatal development is particularly important as the dynamic transitions in hematopoiesis during this time may establish a unique environment that is susceptible to injury and inflammation. Since the liver and spleen are believed to contribute to hematopoiesis in the setting of perinatal liver inflammation, it is important to understand the kinetics of neonatal emergency hematopoiesis to highlight the possible mechanisms by which myeloid populations may contribute to liver injury and repair. Insights gained into perinatal liver inflammation mechanisms will undoubtedly lead to tailored therapies for infants and children who suffer from the devastating consequences of perinatal liver injury.
